# Emotional Intelligence May Be Associated with Some Forms of Creative Potential

**DOI:** 10.3390/jintelligence12120121

**Published:** 2024-11-26

**Authors:** Ahmed M. Abdulla Alabbasi, Mark A. Runco, Abed Al-Nasser D. Al Jarrah, Nada A. Aljohani, Alaa Eldin A. Ayoub

**Affiliations:** 1 Department of Special Education, Arabian Gulf University, Manama P.O. Box 26671, Bahrain; abedaj@agu.edu.bh (A.A.-N.D.A.J.); nadaahj@agu.edu.bh (N.A.A.); 2 Creativity Research & Programming, Southern Oregon University, Ashland, OR 97520, USA; runcom@sou.edu; 3 Radical Creativity, Aalto University, Otakaari 1, FI-02150 Helsinki, Finland; 4 Department of Counselling and Educational Psychology, Yarmouk University, Irbid P.O. Box 21163, Jordan; 5 Department of Educational Psychology, Aswan University, Aswan P.O. Box 81528, Egypt; alaaeldinaa@agu.edu.bh

**Keywords:** divergent thinking, emotional intelligence, Social Game test, giftedness

## Abstract

The current study examined the relationship between creative potential, estimated with tests of divergent thinking (DT), and emotional intelligence (EI). Previous research has hinted at a relationship, but the EI–DT relationship may differ as a function of the tasks and the specific components of EI. With this in mind, the present investigation compared two DT tests (Social Games vs. Titles Games) and examined whether or not the Interpersonal and Intrapersonal subscales of EI were more associated with DT than the Adaptability and Stress Management EI subscales. The youth version of the Bar-On Emotional Quotient Inventory (EQ-i: YV) was used to measure EI. The measure of EI and the two DT tests were administered to 244 male and female gifted (*N* = 125) and nongifted (*N* = 119) high school students in Saudi Arabia. The first objective was to examine whether the EI–DT relationship differs based on the nature of the task of the two DT tests used in the current study (Social Games vs. Titles Games). The second objective was to test whether the Interpersonal and Intrapersonal subscales of EI are more associated with DT than the Adaptability and Stress Management EI subscales. Canonical correlation analysis showed that the relationship between the Social Games test and EI was stronger than the relationship between the Titles Games test and EI. Two path analyses were run: one for the total sample and the second for the gifted sample. The likelihood ratio test showed that the Social Games test was more associated with EQ-i subscales than the Titles Games test for both samples. As expected, the Inter- and the Intrapersonal subscales of the EQ-i were more highly related to Social Games fluency and originality scores compared with the Stress Management and Adaptability subscales. Limitations and future directions are discussed.

## 1. Introduction

Arguably, the key issue in early studies of creativity concerned its relationship with intelligence. One perspective was that creativity is just a particular kind of intelligence and may depend on it (e.g., Getzels and Jackson 1962). Empirical work fairly quickly refuted this view. Indeed, ever since Wallach and Kogan (1965a, 1965b), creativity and intelligence have been viewed as distinct. Not entirely distinct, however. They may be related at lower levels of intelligence. Guilford (1968) referred to this as *triangular theory* because bivariate scatterplots of creativity and intelligence data formed a triangle without much dispersion at low levels of intelligence but quite a bit of variability at the upper levels. This implies that, at the upper levels of intelligence, an individual may be creative, but it is far from guaranteed. This view is also described by threshold theory, named because data suggested that there is a minimum level (or threshold) of intelligence that is necessary for creative performances (Runco and Albert 1987). Various empirical efforts, including meta-analyses, have supported threshold theory (Gerwig et al. 2021; Kim 2005), although the actual relationship between creativity and intelligence depends a great deal on what measures are used to quantify each (Runco and Albert 1987).

More broadly, the creativity–intelligence relationship depends on the underlying definitions, given that both “intelligence” and “creativity” have been defined in diverse ways. The *standard definition of creativity* (Runco and Jaeger 2012) points to two requirements: originality and utility. An idea or solution must be unusual or novel (originality) and appropriate, and in some way effective. The current study examined the relationship between creative potential, estimated with tests of divergent thinking (DT), and one particular kind of intelligence, namely emotional intelligence (EI). Two DT tests were used, one commonly used to estimate creative potential, and the other having a focus on social problems. The idea here was that the latter might be more strongly associated with EI than a more general test of DT. The relationship between DT as a construct and EI was not examined here because that was already investigated by Xu et al. (2019); instead, the focus of the current study was whether the DT–EI relationship would differ based on the *nature* of the DT test (i.e., Social Games vs. Titles Games). Along similar lines, a second objective of this investigation was to examine whether or not DT tests used in the current study would be more highly related to some EI subscales than others. In sum, one hypothesis was that the relationship would depend on the DT test type, and the second hypothesis was that the DT–EI relationship would differ based on EI subscales. These hypotheses were suggested by previous research, which is summarized below. 

### Emotional Intelligence and Creativity

The relationship between creativity and emotions has been studied for decades (e.g., Isen 1999; Kaufmann and Vosburg 1997), but interest seems to be on the rise (Hoffmann et al. 2021; Ivcevic and Hoffmann 2019; Ivcevic et al. 2023; Newton 2013; Sundquist and Lubart 2022). The general conclusion is that creativity and emotions, although distinct in several ways, are not totally independent (Storbeck and Clore 2007). There are important implications of this conclusion. A highly creative student might, for example, not perform well on a DT test, not because of his or her low DT skills, but perhaps because of test anxiety or the fear of being less creative than his or her peers. Ivcevic and Hoffmann (2019) recently demonstrated that emotions can fuel creativity. Two meta-analyses reported that positive mood can enhance creativity (Baas et al. 2008; Davis 2009). 

EI in particular might be related to creativity. For instance, the Interpersonal component of EI (one of the core components examined in the current study) may be related to creativity when an individual must convince others about his or her creative idea or product (i.e., persuasion; Simonton 1995). Further, EI and creativity may each require some degree of Adaptability, which is an essential element of EI and creativity (Bar-On 2006; Cohen 1989; Kim and Pierce 2013; Petrides et al. 2007; Sternberg 2019). The current study measures the mixed ability EI model defined as “a cross-section of interrelated emotional and social competencies, skills, and facilitators that determine how effectively we understand and express ourselves, understand others and relate with them, and cope with daily demands and challenges” (Bar-On 2006, p. 563). This approach differs from the performance-based EI model (Mayer et al. 2000). Matthews et al. (2002) reported five differences between these two models: (a) typical performance vs. maximal performance, (b) internal appraisal of performance vs. external appraisal of performance, (c) great response bias vs. minimal response bias, (d) short vs. long administration time, and (e) personality like vs. ability like (see Table 5.1, p. 180).

Previous research has investigated the relationship between DT and EI (Ferdowsi and Razmi 2022; Geher et al. 2017; Giancola et al. 2024; Şahin 2016; Sánchez-Ruiz et al. 2011; Sordia et al. 2019; Tong et al. 2022; Tu et al. 2020). Perhaps the most comprehensive work in this area was a meta-analysis conducted by Xu et al. (2019), which reported a moderate relationship between creativity and EI (*r* = 0.32). This overall effect size was, however, based on EI and varied measures of creativity, such as creative personality, creative behavior, creative product, and DT. Individual studies on the relationship between EI and DT have shown mixed results. This probably reflects the different EI and DT assessments used, different indices of DT, task modality (figural vs. verbal DTs), and other factors such as gender, age, and culture. Xu et al. (2019) did not examine DT-related factors (i.e., test type and DT indices), nor did they examine the difference in the relationship between EI and DT, looking at specific EI assessment type (i.e., ability EI vs. trait EI). The current study re-examined the relationship between EI, measured by the Bar-On Emotional Quotient Inventory Youth Version (Bar-On and Parker n.d.), and creative potential, as measured by two DT tests. One DT test was Titles, which has been called the best test of DT (Guilford 1968; Runco et al. 2016). It has not been used previously in research on EI. DT in previous research on EI used the Alternative Uses Test or the Torrance Tests of Creative Thinking (Xu et al. 2019). The other DT test employed here was a relatively new assessment. It was chosen because it would seem to require EI (or some components of it), such as the Interpersonal and Intrapersonal subscales. It assesses individuals’ ability to think about social problems and generate as many ideas as possible for these problems. 

There is evidence that different DT tests elicit different performances (Erwin et al. 2022; Runco et al. 2016), which explains the hypothesis that the DT–EI relationship will be affected by the DT measure used. The same applies to the EI measure used (Abdulla Alabbasi et al. 2021; O’Connor et al. 2019; Zeidner et al. 2005). Zeidner et al. (2005) examined differences between gifted and nongifted students using two different EI tests: (a) the Mayer–Salovey–Caruso Emotional Intelligence Test (MSCEIT) and (b) the Schutte Self-Report Inventory (SSRI). Zeidner et al. (2005) found that gifted students scored higher on the MSCEIT but lower on the SSRI than their nongifted peers. In a meta-analysis on the difference between gifted and nongifted students in EI, Abdulla Alabbasi et al. (2021) found that gifted students outperformed nongifted students, and the EI measures were a significant moderator.

This is the first investigation to examine the specific components of EI (Interpersonal and Intrapersonal subscales) as they may be related to two creative potential tests. The main hypothesis was that the relationship between the Interpersonal and Intrapersonal subscales of the EI, with the Social Games test, would be stronger than those using other EI subscales (i.e., Adaptability and Stress Management) because of (a) the task nature of the Social Games and (b) research sometimes showing only moderate correlations between Interpersonal and Intrapersonal subscales (0.39 to 0.75; Ghenaati and Naeini 2019; Tommasi et al. 2023). A second objective also confirms that the present study offers a unique contribution to the creativity and EI literature. This involved taking giftedness status into account in the analyses of the relationship between DT and EI. The rationale here was that recent evidence showed that gifted students are more emotionally intelligent than their nongifted peers (Abdulla Alabbasi et al. 2021; Ogurlu 2021) and are more creative than nongifted students (Abdulla Alabbasi et al. 2024; Dereli 2023; Kahveci and Akgul 2019). With few exceptions (Chan 2005; Sahin et al. 2016; Şahin 2016; Sanchez and Blanc 2023), the EI–DT relationship was studied with gifted samples, and none of these works examined the difference in the EI–DT relationship based on giftedness status. A third novel contribution of the current investigation was its use of canonical correlation and path analysis to compare the association between EI and two tests of creative potential for the total sample and the gifted sample. This statistical approach minimizes the possibility of Type 1 errors. In sum, the present research addressed the following questions:

- Would the EI–DT relationship differ based on the nature of the task of the two DT tests used in the current study (Social Games vs. Titles Games)?
- Would the Interpersonal and Intrapersonal subscales of EI be more associated with DT than the Adaptability and Stress Management EI subscales?

## 2. Method

### 2.1. Participants and Procedures

The sample comprised 244 male and female high school students in Saudi Arabia in the tenth, eleventh, and twelfth grades (*M_age_* = 15.74; SD = 0.93). The sample included both gifted (*N* = 125; 54 boys and 71 girls) and nongifted (*N* = 119; 63 boys and 56 girls) students. Since education in Saudi Arabia is segregated by gender, a male author collected data from boys’ schools, and a female researcher collected data from girls’ schools. Participants were recruited randomly from two high schools in the Northern region of Saudi Arabia after obtaining approval from the General Directorate of Tabuk City, Ministry of Education (Approval ID: 4400753371; date of approval: 23 January 2023), and consent from their parents. The involvement in the study was voluntary, and there was no credit for participation. The gifted and the nongifted students were recruited from the same schools since the gifted education program in Saudi Arabia is based on a pull-out method (Ayoub et al. 2022). Both groups study together; the only difference is that gifted students receive enrichment programs/classes (i.e., pull-out) in the area where they show exceptionality (i.e., science, math, languages, etc.). According to the National Center for Assessment in Saudi Arabia, the first step in the identification process is a self *or* a teacher nomination. The student/teacher registers in a special online portal for both types of nominations. The nomination begins in October of each academic year. The following four assessments are administered for the final selection of gifted learners: (a) a mental flexibility test, (b) a scientific and mechanical reasoning test, (c) a mathematical and spatial reasoning test, and (d) a linguistic reasoning and reading comprehension test. All of the assessments were developed and normed for use in Saudi Arabia. Those who score at or above the 95th percentile in at least two tests (and above the 90th percentile in the third) are selected for the gifted program (for more details on the identification process in Saudi Arabia, see Abdulla Alabbasi et al. 2024).

A booklet with a unique code was printed for each participant. The first page included the consent form to be signed by students, followed by (a) the demographics page (age, grade, sex, parents’ education, birth order, and family size), (b) the Titles Games test, (c) the Bar-On Emotional Quotient Inventory: Youth Version (Bar-On EQ-i: YV), and (d) the Social Game test.

The tests were administered in the second class/session for consistency in both the boys’ and girls’ schools. The authors/data collectors were available to answer any questions before the start of the test session. All DT tasks were untimed, and “be fluent and creative” instructions were emphasized (see the Section 2). This follows from the evidence that untimed tests best support originality (Paek et al. 2021; Wallach and Kogan 1965b) and that explicit instructions result in higher DT performance (Acar et al. 2020; Said-Metwaly et al. 2020). The Bar-On EQ-i: YV was also administered under an untimed condition. The average time for completing the test sessions was 43.73 min.

### 2.2. Instruments

*Titles Test*. The Titles test was administered to assess participants’ fluency and originality. The Titles test was developed by Guilford (1968) and has been used in several recent studies (Runco and Abdulla Alabbasi 2024; Runco et al. 2016). Runco and Abdulla Alabbasi (2024) reported a predictive validity of 0.73 between Titles and the *Creative Activity and Accomplishment Checklist*. Runco and Abdulla Alabbasi (2024) reported reliability coefficients of 0.84 for fluency, 0.81 for flexibility, and 0.79 for originality. The original version of Titles gave a paragraph and asked respondents to list possible titles. The newer version presents the title of a famous book or movie, and respondents are asked to list optional titles. To ensure that all participants in the present research were familiar with the titles used in each of the three tasks, three classes representing 10th, 11th, and 12th-grade male and female students from the same schools were asked to rate 10 movies with which they were the most familiar (1 being more familiar and 10 being less familiar). The task was simple: indicate the best movies from 1 to 10. The questionnaire was distributed using Google Forms. After collecting all responses, the top three frequent movies were: Toy Story, Harry Potter, and Star Wars. The directions for the Titles test were as follows: “List alternative titles for the movies below. Spelling does not matter, and there are no grades for this. Have fun and list as many alternatives as you can.”

Titles was scored for fluency and originality. Fluency is defined as the number of unique and unrepeated ideas related to the stimuli. Originality is defined in terms of the statistical infrequency of responses related to the task (Runco 1991). The present study scored originality using a 1% cut-off, so ideas given by 1% of the sample or less contributed to the originality score. 

*The Social Games*. Runco developed the Social Games Test as part of the Runco Creativity Assessment Battery (r-CAB). It asks participants to generate options for various social situations. Each task is open-ended and thus allows scoring like other DT tests. It is categorized as a realistic test of DT because the social situations are things that could occur in the natural environment. Runco et al. (2024), for example, used Social Games along with other realistic tests of DT in their comparison of different GAI platforms. That previous project did not have human subjects (only GAI platforms), so there is no previous reliability; but of course, we report the reliability of Social Games below, in the Results. 

Three Social Games activities were used in the current study. Participants were asked, one at a time, to list (a) different ways of conveying to someone that they are not dressed appropriately, (b) different ways to convey the idea that the meal your friend just prepared for you does not taste good (in fact, it tastes bad!), and (c) different ways for conveying the idea that someone’s performance (say, in a sport, or on a test) was very poor. The verbatim directions for Social Games were as follows:

“Sometimes, we must find a polite way to say things to others. This allows you to change how you say something so that it is socially acceptable. You will be given a blunt expression (“you have body odor”) and should list as many different ways of conveying that idea to someone–but using different wording and perhaps nuance, euphemism, or simply ambiguity. There are no grades or points, and spelling does not matter. This is not a test; it is a game. The objective is to list as many different ways as possible to convey the target idea. Instead of saying, “you have body odor”, you might say “do you smell something?” or “Have you been working out?” Or have you tried that new high-tech deodorant?” There are many ways of expressing the target idea, and the most you list, the better!”

*The Bar-On Emotional Quotient Inventory: Youth Version (Bar-On EQ-i: YV)*. The current study used a short youth version of the EQ-i. The youth version of the EQ-i was designed for individuals from 7 to 18 years old and it assesses four subskills: (a) adaptability, (b) interpersonal skills, (c) intrapersonal skills, and (d) stress management. The EQ-i is a self-report mixed-model assessment of EI. Bar-On and Parker (n.d.) reported the reliability of the short youth version of the EQ-i, which ranged between 0.65 and 0.87. Many studies demonstrated the validity and the reliability of the EQ-i in different cultures (Al-Hamdan et al. 2017; Bar-On and Parker n.d.; Esnaola et al. 2018a, 2018b; Navarro-Roldán et al. 2023; Stanimirovic and Hanrahan 2012). The EQ-i was translated and normed in different Arab countries, including Bahrain, Lebanon, and Jordan (Al-Hamdan et al. 2017; Al-Nabhan 2008; El Hassan and El Sader 2005). Participants rate each item on a 4-point Likert scale ranging from 1 (very seldom true of me) to 4 (very often true of me). 

Table 1 shows Cronbach’s Alpha and McDonald’s Omega reliability coefficients.

## 3. Results

### 3.1. Differences Between Gifted and Nongifted Students in DT

Before testing our primary research question of the current investigation, it was reasonable to examine the differences between gifted and nongifted students on the two DT tests, especially since the Social Games test is being used for the first time with human subjects. Four one-way analyses of variance (ANOVAs) were run to test whether gifted and nongifted students’ performance on the Titles Games differed from performance on Social Games. A Bonferroni correction was used to control for type I error; thus, the significance level was 0.0125 (0.05/4). The results showed that gifted students outperformed their nongifted peers in (a) Social Games fluency, *F*(1, 242) = 148.76, *p* < .001, η_p_^2^ = 0.381; (b) Social Games originality, *F*(1, 242) = 73.13, *p* < .001, η_p_^2^ = 0.232; (c) Titles Games fluency, *F*(1, 242) = 113.29, *p* < .001, η_p_^2^ = 0.319; and (d) Titles Games originality, *F*(1, 242) = 21.83, *p* < .001, η_p_^2^ = 0.083.

Four paired-sample *t*-tests were run to test which DT test elicits more fluency and originality. Again, a Bonferroni correction was used to control for type I error with a significance level of 0.0125. For the gifted sample, the results showed that the Social Games elicited more ideas (fluency) than the Titles Games, *t*(124) = 42.58, *p* < .001, *d* = 2.38, and more unique ideas (originality) than the Titles Games, *t*(124) = 46.92, *p* < .001, *d* = 1.69. The same pattern was observed with the nongifted sample where the Social Games test elicited more ideas (fluency), *t*(118) = 42.58, *p* < .001, *d* = 2.15, and more unique ideas (originality), *t*(118) = 59.08, *p* < .001, *d* = 2.31. Table 2 shows descriptive statistics.

### 3.2. The Relationship Between Social Games Test and EQ-i

The primary research question of the current investigation is whether the Social Games test, a DT test that was developed to assess DT in the interpersonal domain, would be more highly related to EI than the Titles Games. Table 3 shows the correlations between EI subscales, total EQ-i, and the two DT tests. The correlations between Social Games fluency and EI ranged between −0.04 (for Stress Management) and 0.22 (for the total EQ-i). The correlations between Social Games originality and EI ranged between 0.03 (for Stress Management) and 0.17 (for the total EQ-i). The correlations between Titles Games fluency and EI ranged between −0.03 (for Stress Management) and 0.23 (for Intrapersonal and total EQ-i). The correlations between Titles Games originality and EI ranged between −0.02 (for Stress Management) and 0.20 (for Adaptability). The highest correlation between EI subscales was 0.63 between Inter- and Intrapersonal subscales, and the lowest was 0.39 between Interpersonal and Stress Management subscales.

Two types of analyses were performed to answer the main research question: (a) the canonical correlation, which was used to test the overall relationship between the four EQ-i subscales and the two DT tests, and path analysis, which offers more detailed information about the association between the two DT tests used in this study and each of the EQ-i subscales.

#### 3.2.1. Canonical Correlation Analyses

Two canonical correlation analyses were run to test whether or not Social Games fluency and originality were more highly related to EI than fluency and originality from the Titles Games. The first canonical correlation analysis included fluency and originality scores in the Social Games test and the four EI subscales. Two orthogonal variates were uncovered [*Rc* = 0.275 and 0.203, Wilks = 0.886, (*F*(8, 476) = 3.70, *p* < .001, for the first variate; 0.959 (*F*(3, 239) = 3.42, *p* = .018 for the second variate]. The second canonical correlation analysis included fluency and originality scores in the Titles Games test and the four EI subscales. Again, two orthogonal variates were uncovered. The first variate was statistically significant, *Rc* = 0.239, Wilks = 0.937, (*F*(8, 476) = 1.97, *p* = .049, while the second variate was not statistically significant, *Rc* = 0.079, Wilks = 0.994, (*F*(8, 476) = 0.51, *p* = .678. These results suggest that the Social Game is more highly related to EI than the Titles Games test (i.e., 0.275 vs. 239). 

#### 3.2.2. Path Analysis

The path models tested in this study can be summarized as follows: Model 1 included the data from the total sample to examine the relationship between the scores from Social Games and the EQ-i subscales; Model 2 also used the total sample but examined the relationship between the scores from the Titles Games and the EQ-i subscales; Models 3 and 4 were designed to analyze data from the gifted students’ sample, with Model 3 examining the association between Social Games and EQ-i subscales, and Model 4 examining the association between the Titles Games and EQ-i subscales.

**Figure 1:**
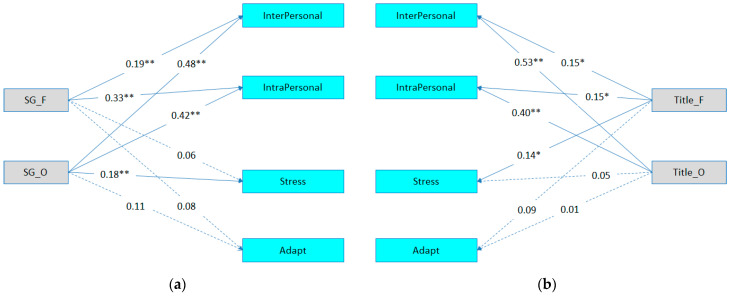
Path analysis models for the study variables (total sample; *N* = 244). (**a**) Model 1: the path analysis between the Social Games test and the EQ-i subscales and (**b**) Model 2: the path analysis between the Titles Games test and the EQ-i subscales. *Note*. SG = Social Games test; Title = Title Game test; F = fluency; O = originality; Stress = stress management; Adapt = adaptability. * *p* < .05. ** *p* < .01.

The models were tested in the path analysis using the LISREL program (Jöreskog and Sörbom 1993). The maximum likelihood method was used to estimate the parameters of the structural equation model. Goodness-of-fit indices for the four models are presented in Table 4. Regarding the total sample, Model 1 indicated that Social Games fluency was significantly associated with (a) Interpersonal EI (β = 0.19, *p* < .01) and (b) Intrapersonal EI (β = 0.33, *p* < .01). There were no significant associations between Social Games fluency and Stress Management nor the Adaptability subscales. Regarding the association between Social Games’ originality and EQ-i subscales, the results indicated that originality was significantly associated with (a) Interpersonal EI (β = 0.48, *p* < .01), (b) Intrapersonal EI (β = 0.42, *p* < .01), and (c) Stress Management (β = 0.18, *p* < .01) subscales. There was a nonsignificant association between originality and Adaptability (see Table 5). Model 2 shows the path analysis results between the Titles Games test (fluency and originality) and the EQ-i subscales. The results indicated that the Titles Games fluency was significantly associated with the (a) Interpersonal EI (β = 0.15, *p* < .05), (b) Intrapersonal EI (β = 0.15, *p* < .05), and (c) Stress Management (β = 0.14, *p* < .05) subscales. The association between Titles Games fluency and Adaptability was nonsignificant. The Titles Games originality was significantly associated with (a) Interpersonal EI (β = 0.53, *p* < .01) and (b) Intrapersonal EI (β = 0.40, *p* < .05). The association between Titles Games originality and both Stress Management and Adaptability were nonsignificant. Given that the number of independent and dependent variables is the same, and to facilitate a comparison between models M1 and M2, constraints were imposed on M2 by constraining the path between Social Games originality and the Interpersonal subscale. Constraining significant paths can enhance the model fit by reducing the degrees of freedom, which, in turn, may improve the interpretability and stability of the model. However, it is important to note that constraining parameters can be helpful when dealing with parameters that exhibit excessive variance, ensuring a more stable and reliable estimation process. This approach aligns with the principles discussed in Byrne (2016) and Kline (2016), which emphasize the importance of appropriately applying constraints to improve model quality while maintaining theoretical coherence. The likelihood ratio test (LRT) showed that Social Games (Model 1) was more strongly associated with EQ-i subscales than the Titles Games test (Model 2), (Δ*x*^2^ = 4.39, Δdf = 1, *p* = 0.36) (See Figure 1).

**Figure 2:**
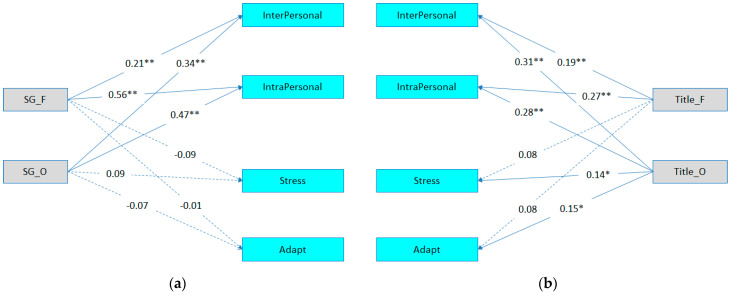
Path analysis models for the study variables (gifted sample; *N* = 125). (**a**) Model 3: the path analysis between the Social Games test and the EQ-i subscales and (**b**) Model 4: the path analysis between the Titles Games test and the EQ-i subscales. *Note*. SG = Social Games test; Title = Titles Games test; F = fluency; O = originality; Stress = stress management; Adapt = adaptability. * *p* < .05. ** *p* < .01.

Models 3 and 4 were designed to examine the association between the Social Games and Titles Games test, and EQ-i for the gifted sample. The results indicated that Social Games fluency was significantly associated with (a) Interpersonal EI (β = 0.21, *p* < .01) and (b) Intrapersonal EI (β = 0.56, *p* < .01). The associations between Social Games fluency and Stress Management and Adaptability subscales were nonsignificant. Regarding the association between Social Games’ originality and EQ-i subscales, the results indicated that there was a significant association of originality with (a) Interpersonal EI (β = 0.34, *p* < .01) and (b) Intrapersonal EI (β = 0.47, *p* < .01). Again, the associations between Social Games originality and both Stress Management and Adaptability subscales were nonsignificant. Finally, Model 4 examined the Titles Games test (fluency and originality) and the EQ-i subscales. Results indicated that the Titles Games fluency was significantly associated with (a) Interpersonal EI (β = 0.19, *p* < .01) and (b) Intrapersonal EI (β = 0.27, *p* < .01). The associations between fluency and both Stress Management and Adaptability were not statistically significant. Finally, originality from the Titles Games test was significantly associated with the (a) Interpersonal EI (β = 0.31, *p* < .01), (b) Intrapersonal EI (β = 0.28, *p* < .01), (c) Stress Management (β = 0.14, *p* < 0.05), and (d) Adaptability (β = 0.15, *p* < .05) subscales. Again, to facilitate a comparison between models M3 and M4, constraints were imposed on M4 by constraining the path between Social Games originality and the Interpersonal subscale. As was the case with the total sample, the LRT showed that Social Games (Model 3) was more strongly associated with the EQ-i subscales than the Titles Games test (Model 4), (Δ*x*^2^ = 3.88, Δdf = 1, *p* = .049) (See Figure 2).

## 4. Discussion

The current investigation had two specific objectives. The first concerned a possible difference whereby one test of DT (Social Games) would be more strongly related to the Inter- and Intrapersonal EI scales than would the other DT test (i.e., Titles). This follows from the fact that the Social Games measure has an interpersonal emphasis, as does the Interpersonal subscale of the EQ-i measures. There was no specific hypothesis about the relationship between the Social Games test and other EI subscales. Previous research has demonstrated that the Inter- and Intrapersonal EI subscales are moderately to highly correlated (Ghenaati and Naeini 2019; Tommasi et al. 2023). This was the case in the current study as well. Table 3 shows that the highest correlation between EI subscales was between the Inter- and Intrapersonal subscales (*r* = 0.63). 

The results from the bivariate correlation showed mixed results regarding the different relationships of Social Games and Titles with EI. Social Games fluency and originality were more strongly correlated with the Interpersonal subscale than scores from Titles fluency and originality, and Titles fluency and originality were more strongly correlated with the Intrapersonal subscale than were Social Games fluency and originality. In every case, the magnitude of the difference was (*rs* = 0.19 vs. 0.16, 0.12 vs. 0.10, 0.23 vs. 0.20, and 0.16 vs. 0.13, respectively). That being said, the canonical correlation and path analyses showed that the Social Games test was more strongly associated with EI than the Titles Games test. The path analysis results indicated that the association between Social Games fluency and originality, on the one hand, and Inter- and Intrapersonal subscales, on the other hand, was higher than the association between the other two scales, at (βs 0.19 to 0.33) and (βs 0.42 to 0.48), respectively. The Social Games outperformed Titles only clearly when modeled simultaneously. This finding is interesting from a theoretical perspective. From an applied diagnostic perspective, however, the two tests did not differ in their relationship with EI, when applied on their own, as is apparent in the bivariate relations. A similar conclusion was reached for the Titles Games fluency and originality, at (β 0.15) and (βs 0.40 to 0.53), respectively. When only a gifted sample was used, the results were much the same. For Models 1 and 2, there were two exceptions: (a) the association between Social Games originality and Stress Management was statistically significant (β *=* 0.18), and (b) the association between Titles Games fluency and Stress Management was statistically significant (β *=* 0.14). Finally, when only gifted students were considered (i.e., Models 3 and 4), the Titles Games originality and both Stress Management and Adaptability were statistically significant, at (β = 0.14) and (β = 0.15), respectively. Future research might investigate the relationship between DT and both Stress Management and Adaptability.

The second objective was to investigate the relevance of giftedness status on the DT–EI relationship. All students, gifted and nongifted, scored higher on the Social Games test than the Titles Games test (see Table 2). The canonical correlation between EI and DT showed that the relationship between Social Games and EI was higher than the relationship between Titles Games and EI (*Rc* = 0.275 vs. 0.239). The path analysis confirmed that the EI–DT association was higher for the Social Games vs. Titles Games tests in both the total sample (Models 1 and 2) and the gifted sample (Models 3 and 4). As discussed in the Introduction, the current study assessed the mixed ability EI model as measured by the EQ-i. Future research should investigate the relationship between DT and an ability-based EI measure to determine if such a relationship would differ based on the measure used.

There are limitations of this research, as well as suggestions for future directions. First is the limitation of relying on one measure of EI. Some research shows that the mixed-model measures of EI, which are self-report-based, differ from ability-based EI assessments (Abdulla Alabbasi et al. 2021, 2023; Alabbasi et al. 2023; Goldenberg et al. 2006; Zeidner et al. 2005). Goldenberg et al. (2006) compared performance-based vs. self-report-based EI assessments and concluded that these two types of measures were unrelated. Still, performance-based EI assessments have some limitations, which are discussed in detail in MacCann et al. (2003). Future research might compare the EI–DT relationship using performance-based EI assessments. The second limitation is that the findings might only generalize to academically gifted students. The EI–DT relationship might vary in different populations of gifted learners, such as the artistically and musically gifted, or academically gifted students in specific domains. The third limitation is that Cronbach’s alpha coefficient for originality in Social Games was below 0.70. Then again, McDonald’s omega coefficient was 0.76, which is adequate. In addition, alphas around 0.70 are not uncommon in creativity research. This is probably due to the fact that, unlike convergent thinking tests (e.g., IQ and standardized achievement tests), measures of DT do not have a single answer. This leads to more variety and dispersion. Also, Acar et al. (2024) reported a reliability generalization meta-analysis of the Torrance Test of Creative Thinking, which is a well-known measure of DT. It included 44 studies. The reliability (Omega) ranged between 0.62 and 0.85. The fourth limitation was that although the results showed differences among means, some were marginal. The differences uncovered may have practical significance in the context of research related to human behavior and psychology. However, the study does not provide conclusive evidence on the magnitude of the effects. Results might be viewed as indicative of an exploration of trends of the differences, within this specific framework. Certainly, future studies with larger sample sizes would be useful to confirm differences and replicate the current findings. Similarly, future research could test cultural differences in the EI–DT relationship since much evidence shows that EI performance differs between cultures and that EI is culture-specific (Abdulla Alabbasi et al. 2023; Gunkel et al. 2014; Huynh et al. 2018; Pathak and Muralidharan 2020). Finally, future research could compare models that use identical variables and samples to reach more reliable conclusions.

We conclude with two practical implications. First is that the Social Games test can be used to identify gifted students in social domains. This is suggested by the fact that the ANOVA and *t*-test results showed that gifted and nongifted students elicited more original ideas on Social Games than on Titles. Thus, it was discriminating. Research might include Social Games, then, especially when originality is the concern. Second, and more broadly, the current results suggest that both EI tests and Social Games could be included in identification and support programs, for a deeper understanding of inter- and intrapersonal emotional intelligence. 

## Figures and Tables

**Table 1:** Reliability coefficients of the study variables.

Variables	Cronbach’s Alpha Coefficients	McDonald’s Omega Coefficients
Social Games Fluency	0.84	0.85
Social Games Originality	0.63	0.76
Titles Games Fluency	0.87	0.88
Titles Games Originality	0.79	0.81
Intrapersonal	0.76	0.81
Interpersonal	0.78	0.83
Adaptability	0.84	0.89
Stress Management	0.76	0.81
Total EQ-i	0.84	0.89

**Table 2:** Means and standard deviations for the gifted and non-gifted students.

Variables	Gifted (*N* = 125)		Non-Gifted (*N* = 119)		*F*	*p*	η_p_^2^
	M	SD	M	SD			
Fluency (Titles)	7.76	2.04	5.38	1.38	113.29	<.001	0.319
Originality (Titles)	3.89	0.93	3.41	0.63	21.83	<.001	0.083
Fluency (Social Games)	15.71	3.66	10.57	2.84	148.76	<.001	0.381
Originality (Social Games)	9.98	2.46	7.71	1.57	73.13	<.001	0.232

*Note*: A Bonferroni correction was applied to the four comparisons. It resulted in a significance level of (0.0125); thus, it is safe to conclude that the results presented in Table 1 are not influenced by Type I error.

**Table 3:** Correlations between fluency and originality in Titles Games and Social Games DT tests and EQ-i (*N* = 244).

Variables	Fluency (SG)	Originality (SG)	Fluency (Titles)	Originality (Titles)	Interpersonal	Intrapersonal	Adaptability	Stress Management	Total EQ-i
Fluency (SG)	1	0.47 **	0.42 **	0.39 **	0.19 **	0.20 **	0.19 **	−0.04	0.22 **
Originality (SG)		1	0.23 **	0.25 **	0.12	0.13 *	0.16 *	0.03	0.17 **
Fluency (Titles)			1	0.85 **	0.16 *	0.23 **	0.18 **	−0.03	0.23 **
Originality (Titles)				1	0.10	0.16 *	0.20 **	−0.02	0.19 **
Interpersonal					1	0.63 **	0.55 **	0.39 **	0.83 **
Intrapersonal						1	0.53 **	0.50 **	0.66 **
Adaptability							1	0.52 **	0.77 **
Stress Management								1	0.46 **
Total EQ-i									1

* *p* < .05. ** *p* < .01.

**Table 4:** Goodness-of-fit indices for the different models.

Model	*x* ^2^	*Df*	*x*^2^/*df*	RMSEA 90% CI	CFI	SRMR	NFI
Model_1	10.04	6	1.67	0.053 [0.050, 0.056]	93	0.06	95
Model_2	14.21	5	2.84	0.087 [0.082, 0.092]	93	0.09	92
Model_3	9.18	6	1.53	0.062 [0.059, 0.065]	92	0.07	94
Model_4	13.06	5	2.61	0.084 [0.081, 0.087]	91	0.08	91

*Note*. CI, confidence interval; RMSEA, root mean square error of approximation; CFI, comparative fit index; NFI, Normed Fit Index.

**Table 5:** Path analysis models between Social Games, Titles Games, and EI.

Model	β	SE	*t*	Model	β	SE	*t*
Model-1				Model-2			
SG_F → Interpersonal	0.19	0.072	2.64 **	TG_F → Interpersonal	0.15	0.071	2.11 *
SG_F → Intrapersonal	0.33	0.073	4.52 **	TG_F → Intrapersonal	0.15	0.073	2.05 *
SG_F → Stress Management	0.06	0.057	1.05	TG_F → Stress Management	0.14	0.069	2.03 *
SG_F → Adaptability	0.08	0.058	1.38	TG_F → Adaptability	0.09	0.069	1.30
SG_O → Interpersonal	0.48	0.073	6.58 **	TG_O → Interpersonal	0.53	0.072	7.36 **
SG_O → Intrapersonal	0.42	0.072	5.83 **	TG_O → Intrapersonal	0.40	0.071	5.63 **
SG_O → Stress Management	0.18	0.058	3.10 **	TG _O → Stress Management	0.05	0.070	0.71
SG_O → Adaptability	0.11	0.058	1.90	TG_O → Adaptability	0.01	0.058	0.17
Model-3				Model-4			
SG_F → Interpersonal	0.21	0.064	3.28 **	TG_F → Interpersonal	0.19	0.067	2.84 **
SG_F → Intrapersonal	0.56	0.066	8.48 **	TG_F → Intrapersonal	0.27	0.065	4.15 **
SG_F → Stress Management	−0.09	0.071	−1.27	TG_F → Stress Management	0.08	0.069	1.16
SG_F → Adaptability	−0.01	0.072	−0.13	TG_F → Adaptability	0.08	0.070	1.14
SG_O → Interpersonal	0.34	0.067	5.07 **	TG_O → Interpersonal	0.31	0.068	4.56 **
SG_O → Intrapersonal	0.47	0.068	6.91 **	TG_O → Intrapersonal	0.28	0.065	4.31 **
SG_O → Stress Management	0.09	0.070	1.29	TG_O → Stress Management	0.14	0.068	2.06 *
SG_O → Adaptability	−0.07	0.071	−0.99	TG_O → Adaptability	0.15	0.067	2.24 *

*Notes*. β = standardized path coefficient; SE = standard error; SG = Social Games test; TG = Title Game test; F = fluency; O = originality. * *p* < .05. ** *p* < .01.

## Data Availability

Data is available upon request from the first author.
